# Case Report: Two Distinct Focal Congenital Hyperinsulinism Lesions Resulting From Separate Genetic Events

**DOI:** 10.3389/fped.2021.699129

**Published:** 2021-07-16

**Authors:** Elizabeth Rosenfeld, Lauren Mitteer, Kara Boodhansingh, Susan A. Becker, Heather McKnight, Linda Boyajian, Amanda M. Ackermann, Jennifer M. Kalish, Tricia R. Bhatti, Lisa J. States, N. Scott Adzick, Katherine Lord, Diva D. De León

**Affiliations:** ^1^Division of Endocrinology and Diabetes, Children's Hospital of Philadelphia, Philadelphia, PA, United States; ^2^Congenital Hyperinsulinism Center, Children's Hospital of Philadelphia, Philadelphia, PA, United States; ^3^Department of Pediatrics, Perelman School of Medicine at the University of Pennsylvania, Philadelphia, PA, United States; ^4^Division of Human Genetics, Children's Hospital of Philadelphia, Philadelphia, PA, United States; ^5^Department of Pathology and Laboratory Medicine, Children's Hospital of Philadelphia, Perelman School of Medicine at the University of Pennsylvania, Philadelphia, PA, United States; ^6^Department of Radiology, Children's Hospital of Philadelphia, Perelman School of Medicine at the University of Pennsylvania, Philadelphia, PA, United States; ^7^Department of Surgery, Children's Hospital of Philadelphia, Perelman School of Medicine at the University of Pennsylvania, Philadelphia, PA, United States

**Keywords:** pancreas, beta cells, islets, K_ATP_ channel, hypoglycemia, case report

## Abstract

Focal hyperinsulinism (HI) comprises nearly 50% of all surgically treated HI cases and is cured if the focal lesion can be completely resected. Pre-operative localization of the lesion is thus critical. Few cases of hyperinsulinism with multiple focal lesions have been reported, and assessment of the molecular mechanisms driving this rare occurrence has been limited. We present two cases of multifocal HI, each resulting from two independent, pancreatic focal lesions. ^18^Fluoro-dihydroxyphenylalanine positron emission tomography/computed tomography detected both lesions preoperatively in one patient, whereas identification of the second lesion was an incidental finding during surgical exploration in the other. Complete resection of the focal lesions resulted in cure of the HI in both cases. In each patient, genetic testing of the individual focal lesions revealed different regions of loss of heterozygosity for the maternal 11p15 allele, confirming that each lesion arose from independent somatic events in the setting of a paternally inherited germline *ABCC8* mutation. These cases highlight the importance of a multidisciplinary and personalized approach to the management of infants with HI.

## Introduction

Congenital hyperinsulinism (HI) is the most common cause of persistent hypoglycemia in infants and children (1). Prompt diagnosis and initiation of appropriate treatment are crucial to mitigate risk of permanent hypoglycemic brain damage. Inactivating mutations in the genes encoding the β-cell adenosine triphosphate (ATP)-sensitive potassium (K_ATP_) channel, *ABCC8* (SUR1) and *KCNJ11* (KIR6.2), located on chromosome 11p15.1, are responsible for the most common forms of HI (2). K_ATP_-HI can be classified into two distinct histological forms: a diffuse form, in which all of the pancreatic β-cells are affected, and a focal form of localized islet hyperplasia and dysfunction. While focal HI is more likely to present at an older age and with hypoglycemic seizures than diffuse HI, these two histological forms cannot be distinguished by clinical presentation alone (3). Differentiating between focal and diffuse HI is of paramount importance as surgical excision of the focal lesion is curative. In contrast, the severity of diffuse HI is ameliorated, but not cured, by near-total pancreatectomy, which carries additional risks of diabetes and exocrine pancreatic insufficiency (4).

Focal HI occurs via a “two-hit” mechanism, following the model originally described by Knudson (5), requiring paternal transmission of a recessive loss-of-function mutation in *ABCC8* or *KCNJ11* and a pancreas-limited somatic loss of the maternal 11p15 region compensated by paternal isodisomy (6). Somatic loss of heterozygosity (LOH) leads to imbalanced expression of imprinted genes that regulate cell growth, including *IGF2, H19*, and *CDKN1C*, resulting in the histological findings of focal adenomatous hyperplasia (6). A single paternally inherited recessive K_ATP_ mutation has a 94% positive predictive value for focal HI (2). In these cases, preoperative ^18^fluoro-dihydroxyphenylalanine positron emission tomography/computed tomography (^18^F-DOPA PET/CT) is utilized to localize the focal lesion and guide surgical excision (7). Owing to the mechanism of focal lesion development, the occurrence of multiple focal lesions is exceedingly rare. Few cases of HI with multiple focal lesions have been reported to date (8–12). Of these reports, only one included molecular genetic information confirming that the two lesions, one pancreatic and one ectopic, arose from independent somatic events (8). We describe two cases of HI due to distinct pancreatic lesions which broaden our understanding of the pathogenic mechanisms underlying multifocal HI.

## Materials and Methods

Clinical information was extracted from the electronic medical records as part of a study approved by our Institutional Review Board. ^18^F-DOPA PET/CT was performed as previously described under an Investigational New Drug Application with Food and Drug Administration oversight and Institutional Review Board approval (NCT01916148) (13).

### Genetic Analysis

Genetic testing was performed in CLIA-approved laboratories. DNA extracted from peripheral blood from patient 1 was subjected to polymerase chain reaction (PCR) amplification and Sanger sequencing of the *ABCC8, KCNJ11*, and *GCK* genes, with deletion/duplication analyses for *ABCC8* performed using multiplex ligation-dependent probe amplification. Chromosomal single nucleotide polymorphism (SNP) microarrays were performed on DNA extracted from pancreas tissue from patient 1, and from blood and pancreas tissue from patient 2, using the Illumina CytoSNP850Kv1.1 BeadChip. Whole exome sequencing was performed on DNA extracted from peripheral blood from patient 2 using the xGen Whole Exome Panel kit, and findings were confirmed by Sanger sequencing. In addition, deletion and duplication analysis was performed on peripheral blood DNA from patient 2 by next-generation sequencing for a panel of 23 genes, including *ABCC8*. DNA extracted from pancreas samples from patient 2 underwent PCR amplification and next generation sequencing of the *ABCC8, KCNJ11, GCK, GLUD1, HADH, HNF1A, HNF4A, SLC15A1*, and *UCP2* genes. Methylation analyses of DNA extracted from blood, skin, and pancreas tissue from patient 2 were performed using sodium bisulfite treatment followed by quantitative methylation sensitive PCR (14). The nucleotides of *ABCC8* were numbered according to the sequence reported by Nestorowicz et al. that includes the alternatively spliced exon 17 sequence (NCBI accession no. L78224) (15).

### Histological Analysis

Histology studies were performed on pancreatic tissue obtained at the time of surgery from which formalin-fixed, paraffin-embedded sections were mounted on positively charged slides. Sections were stained with hematoxylin and eosin and primary mouse monoclonal antibodies directed against chromogranin (Dako, clone DAK-A3, 1:200) and p57 (Thermo Scientific, p57^Kip2^ Ab-6, 1:800). Immunohistochemical staining was performed using the Leica Bond Immunohistochemistry Stainer (Leica Biosystems, Inc., Buffalo Grove, IL) following standard protocols and using the Polymer Refine Detection Brown Detection System and DAB Enhancer.

## Case Descriptions

### Patient 1

**Figure 1:**
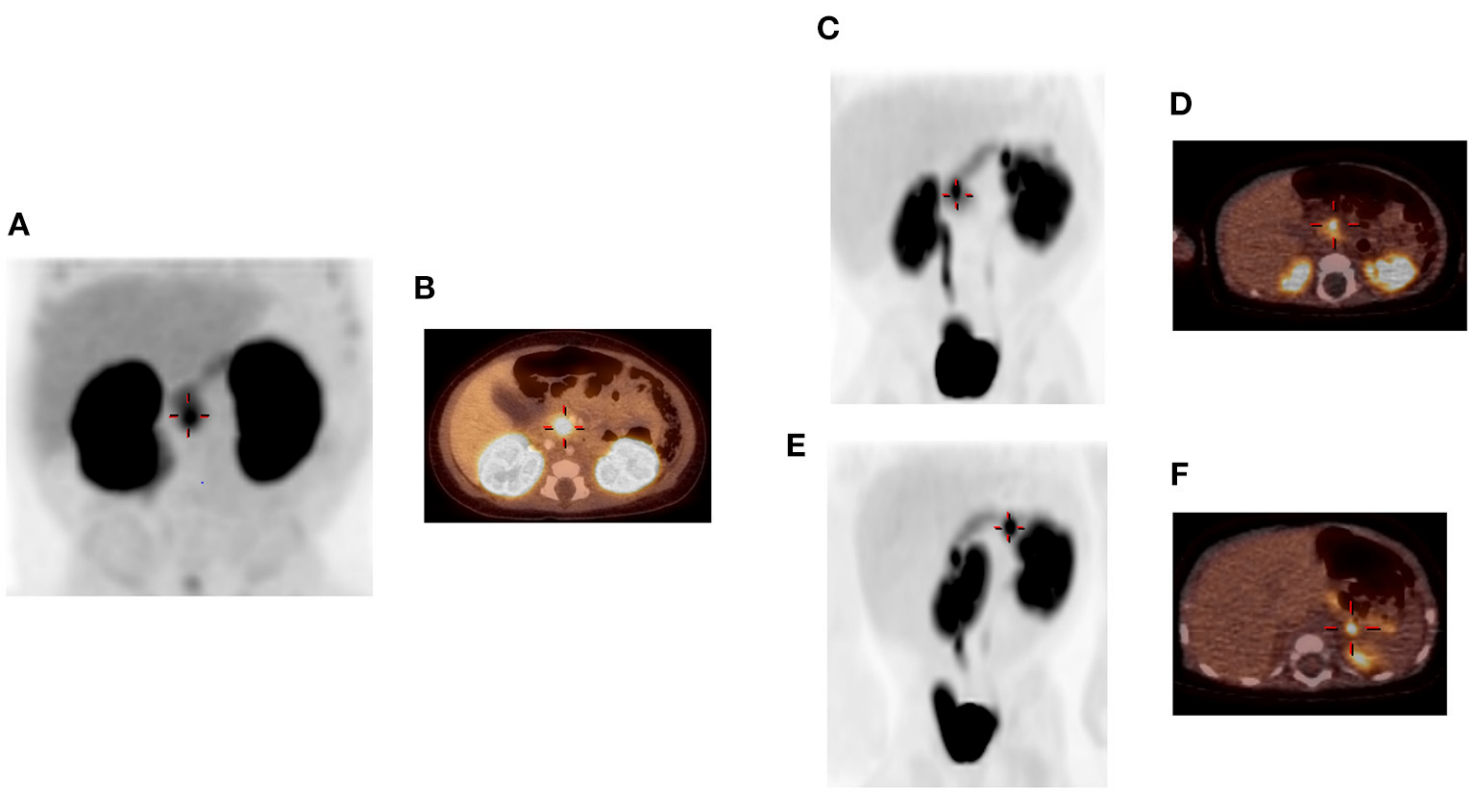
A focal lesion within the pancreatic head/uncinate region (+) is visualized on 3D-MIP image **(A)** and fused PET/CT image **(B)** in patient 1. Two focal lesions are identified in patient 2 **(C–F)**. 3D-MIP image at 50 min **(C)** shows one lesion in the pancreatic head (+) and the other in the proximal pancreatic tail. Fused PET/CT image **(D)** shows the focal lesion in the pancreatic head (+). Rotated 3D-MIP image **(E)** permits better visualization of the proximal tail lesion (+), which is shown on fused PET/CT image **(F)** to be exophytic along posterior margin of pancreas. High uptake in the kidneys and bladder is visualized in all images, as expected, due to renal excretion of the radioisotope. Images were originally published in JNM. States LJ, Davis JC, Hamel SM, Becker SA, Zhuang H. Imaging of congenital hyperinsulinism. J Nucl Med. © SNMMI.

**Figure 2:**
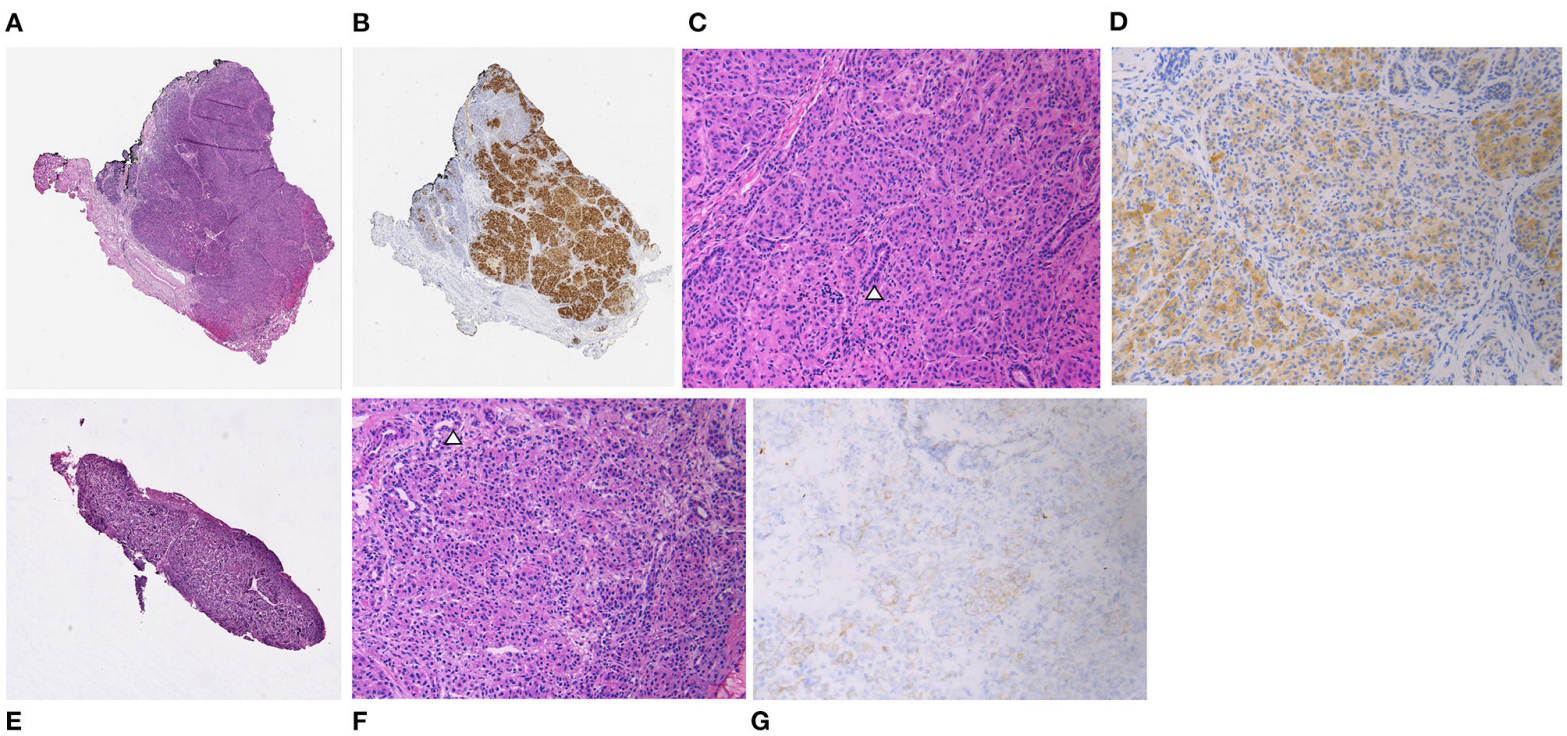
Patient 1. Focal lesions from the pancreatic head [**(A)** hematoxylin and eosin [H&E], **(B)** chromogranin immunohistochemical stain [IHC], both at x20 original magnification] and body [**(E)** H&E, x20 original magnification] composed of localized increases in endocrine tissue with occasional ducts (arrowhead) [**(C,F)** H&E, x200 original magnification]. Endocrine cells demonstrate loss of nuclear immunoreactivity for p57 in both lesions with non-specific reactivity within the cytoplasm [**(D,G)** p57 IHC, x200 original magnification].

An infant male was born at 38 weeks gestation via spontaneous vaginal delivery complicated by nuchal cord. He had Apgar scores of 4 and 9 at 1 and 5 min of life, respectively, and a birth weight of 3.55 kg (66th percentile, appropriate for gestational age). Shortly after birth, plasma glucose was checked due to jitteriness and was found to be 1.55 mmol/L (28 mg/dL). He was admitted to the neonatal intensive care unit where he had repeated episodes of hypoglycemia. Critical specimens confirmed a diagnosis of HI (Table 1), and treatment was initiated with diazoxide and chlorothiazide, without clinical response even after the diazoxide dose was titrated up to 15 mg/kg/day. Genetic testing of peripheral blood DNA of the patient and his parents revealed a single heterozygous paternally inherited recessive *ABCC8* mutation c.3992-9 G>A. ^18^F-DOPA PET/CT, performed to localize the suspected focal lesion prior to surgery, demonstrated increased uptake in the inferior pancreatic head extending into the uncinate region (Figures 1A,B). He underwent 20% pancreatectomy in which a focal lesion in the inferior aspect of the pancreatic head measuring 1 cm in its largest dimension was excised. Intraoperatively, an additional, non-contiguous lesion in the anterior aspect of the proximal pancreatic body measuring 0.4 cm was identified by palpation and excised. Histopathology showed that both resected regions demonstrated localized islet cell hyperplasia with occasional nucleomegaly surrounded by normal pancreas, consistent with focal HI (Figure 2). Chromosomal SNP array analyses performed on the excised pancreatic tissue revealed a 47.74 Mb mosaic region of LOH of 11p15.5p11.2 (chr11:198,510–47,940,925, hg19) in the head lesion and a 34.1 Mb mosaic region of LOH within chromosome 11p15.5p13 (chr11:198,510–34,265,140, hg19) in the body lesion. Concurrent SNP array analysis performed on unaffected pancreatic tissue resulted as normal. The mosaic LOH was consistent with paternal uniparental disomy in the setting of a paternally inherited heterozygous pathogenic variant in *ABCC8* and histologically confirmed focal HI. To assess for resolution of HI, he underwent a fast prior to discharge in which he maintained plasma glucose >3.89 mmol/L (>70 mg/dL) for 9 h with the remainder of results consistent with persistent, mild HI not necessitating additional treatment (plasma glucose 2.44 mmol/L [44 mg/dL] at 21 h of fasting with inappropriately low β-hydroxybutyrate of 1.3 mmol/L and plasma glucose increase of 2.39 mmol/L [43 mg/dL] in response to glucagon administration). Repeat fasting test at 14 months of age demonstrated appropriate rise in β-hydroxybutyrate, undetectable insulin, non-suppressed free fatty acids and insulin growth factor binding protein 1, and lack of glycemic response to glucagon administration consistent with complete HI resolution (Table 1).

**Table 1:** Biochemical evaluation at diagnosis and after surgical removal of the lesions.

	**Patient 1**	**Patient 2**
Maximum glucose infusion rate (mg/kg/min)	15	13.5
**Critical sample at diagnosis**		
Plasma glucose (mmol/L) [mg/dL]	2.05 [37]	2.44 [44]
ß-hydroxybutyrate (mmol/L)	0.19	0.9
Insulin (pmol/L)	118.1	19.44
Free fatty acids (mmol/L)	Not measured	0.38
Δ plasma glucose in response to 1 mg glucagon injection (mmol/L) [mg/dL]	Test not performed	+2.39 [43]
Cortisol (nmol/L)	819.3	524.1
Growth hormone (mcg/L)	23.6	Not measured
**Cure fast**		
Plasma glucose (mmol/L) [mg/dL]	2.89 [52]	2.66 [48]
ß-hydroxybutyrate (mmol/L)	3.4	2.6
Insulin (pmol/L)	<14	<14
Free fatty acids (mmol/L)	1.92	2.62
Insulin growth factor binding protein 1 (nmol/L)	34.9	20.7

### Patient 2

**Figure 3:**
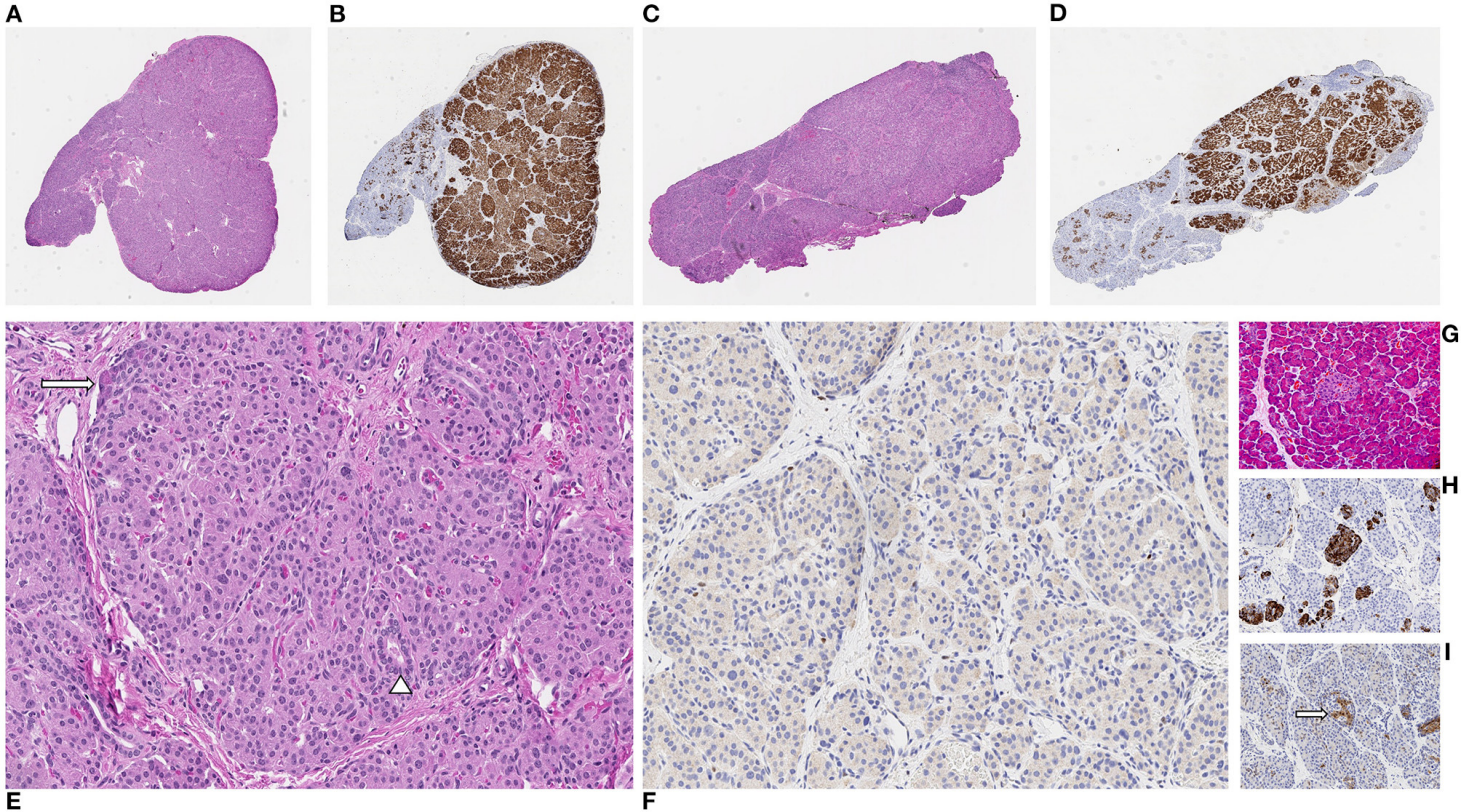
Patient 2. Two distinct focal lesions arising in the pancreatic head **(A,B)** and body **(C,D)** each demonstrating localized increases in endocrine tissue which are highlighted on chromogranin immunohistochemical (IHC) stain [**(A,C)** hematoxylin and eosin [H&E], **(B,D)** chromogranin IHC, all at x20 original magnification]. Higher-power magnification of head lesion demonstrating increased endocrine cells with rare intermixed acinar cells (arrow) and small ducts (arrowhead) [**(E)** H&E, x200 original magnification] with loss of nuclear p57 immunoreactivity within lesional cells [**(F)** p57 IHC, x200 original magnification]. Compared to lesional areas, normal pancreatic tissue shows relatively small islets (arrow) composed of endocrine cells which are positive for chromogranin but with retained nuclear p57 immunoreactivity; non-specific p57 immunoreactivity is observed within the cytoplasm [**(G)** H&E, **(H)** chromogranin IHC, **(I)** p57 IHC, all at x200 original magnification].

Patient 2 was born at 39 weeks gestation. She was delivered via uncomplicated, induced vaginal delivery with Apgar scores of 8 and 9 at 1 and 5 min of life, and birth weight of 4.15 kg (97th percentile, large for gestational age). Hypoglycemia was detected on screening performed shortly after birth and persisted. Diagnostic fasting evaluation confirmed HI (Table 1) that was incompletely responsive to both diazoxide 15 mg/kg/day and octreotide 10 mcg/kg/day. A deletion of unknown significance at chromosomal position chr11:17,416,121–17,416,968 (hg19) including exon 36 of *ABCC8* was detected on peripheral blood genetic testing and was determined to be paternally inherited. In addition, a single base change in intron 16 (c.2222+15 C>A) of the *ABCC8* gene was also identified in peripheral blood DNA; parent of origin was not determined. ^18^F-DOPA PET/CT revealed two separate foci of increased uptake: an exophytic lesion arising from the posterior aspect of the pancreatic body and a lesion on the medial aspect of the pancreatic head (Figures 1C–F). She subsequently underwent 10% pancreatectomy in which a 0.9 cm lesion in the posterior pancreatic body and an 0.8 cm in the pancreatic head were locally excised with clear surgical margins by frozen section. Final histopathology was consistent with focal islet cell hyperplasia (Figure 3). Terminal mosaic regions of LOH of 11p15.5p11.2 of differing lengths were detected on chromosomal SNP array analyses performed on the resected focal lesions. A 48.83 Mb region (chr11:198,510–49,030,187, hg19) was identified within the pancreatic body lesion and a 47.78 Mb region (chr11:198,510–47,976,882, hg19) within the pancreatic head lesion. SNP analysis of chromosome 11 from the unaffected pancreatic tail tissue was normal. Informed by the prior experience with patient 1, methylation studies were performed on DNA isolated from the focal lesion within the pancreatic body. Results further confirmed paternal uniparental disomy of 11p15.5, with loss of methylation at imprinting control region 2 (IC2) and gain of methylation at IC1. DNA isolated from the patient's blood, skin, and unaffected pancreas showed normal methylation at IC1 and IC2. In addition, next generation sequencing was performed on pancreatic samples from both focal lesions as well as surrounding histologically normal pancreatic tissue. These samples were positive for the exon 36 deletion as well as the single base change in intron 16 (c.2222+15 C>A) of the *ABCC8* gene detected in blood. This intronic variant was present at a frequency of 73–86% in affected pancreas suggesting it was on the paternal allele and *in cis* with the exon 36 deletion. Resolution of HI was demonstrated on fasting evaluation prior to hospital discharge (Table 1).

## Discussion

Identification of patients with focal HI is of critical importance, as these patients can be cured if the lesion is localized and fully resected. Over the past several decades, improvements in the diagnosis of focal HI have been bolstered both by advances in molecular diagnostic testing and the implementation of ^18^F-DOPA PET/CT for localization of focal lesions. Focal HI comprises roughly 50% of all surgically treated congenital HI cases (4). In contrast, the presence of multiple focal HI lesions is rare, and few patients with multifocal lesions have been reported to date.

Goossens et al. first reported three cases of “multifocal nesidioblastosis” in 1989 (9). Limited histopathological data is provided, and this report predated our understanding of the genetic and molecular mechanisms responsible for congenital HI. Since then, Ni et al. described a patient with a *KCNJ11* mutation found to have one focal lesion in the pancreatic head and another in the pancreatic body (11). Our group previously reported two separate cases, one suspected and one confirmed, of focal HI in which there were both pancreatic and ectopic intestinal focal lesions (10, 12). In each of these cases, the presence of multiple focal lesions was ultimately identified on ^18^F-DOPA PET/CT imaging, accentuating the utility of this technique in the surgical management of focal HI. However, genetic analysis of the resected tissue was not reported.

To our knowledge, the only report including genetic analyses of tissue from multiple focal lesions is that of Giurgea et al. describing two patients, each with one pancreatic and one ectopic lesion (8). Both patients carried a heterozygous, paternally inherited *ABCC8* gene mutation and were cured after complete lesionectomy. Genotyping of affected tissue by fluorescent PCR assay for chromosome 11p markers revealed somatic deletions of the maternal 11p15 region with different deletion break points within the pancreatic and ectopic lesions. No LOH was detected in samples from unaffected pancreatic tissue.

This report provides a detailed description of multiple focal lesions confined to the pancreas, adding to the limited body of literature on multifocal HI. In both patients presented here, analyses of resected pancreatic tissue revealed two distinct focal lesions with different sizes of LOH within 11p15, confirming that the lesions resulted from distinct somatic events in the setting of a paternally inherited, germline recessive *ABCC8* mutation. The independence of the somatic events leading to multifocal HI is further supported by the distinct anatomic location of the focal lesions identified in patient 1, which reflects independent spatial origin. In pancreas embryogenesis, the inferior pancreatic head and uncinate process arise from the ventral pancreatic bud, and the remainder of the pancreas is formed from the dorsal pancreatic bud (16). In addition, the relative timing of the somatic event may determine focal lesion size (8, 17). Thus, the somatic event leading to the larger focal lesion in the inferior pancreatic head likely occurred within the ventral pancreatic bud earlier in development, whereas the smaller lesion in the pancreatic body may have resulted from a later somatic event within the dorsal bud. These findings augment those of Giurgea et al. and affirm the “two-hit” pathogenetic mechanism of focal HI.

The c.3992-9 G>A recessive mutation in *ABCC8* observed in patient 1 is a well-recognized, founder mutation within the Ashkenazi Jewish population that affects splicing (18). In contrast, the pathogenicity of the *ABCC8* changes detected in patient 2 are not well-established. The paternally inherited heterozygous deletion in exon 36 would be predicted to result in a shift in the mRNA reading frame, resulting in an early termination codon. This would affect the nucleotide-binding fold 2 (NBF2) domain of SUR1, mutations in which represent a common molecular mechanism of diazoxide-unresponsive HI (19). The single base change in intron 16 (*ABCC8*: c.2222+15 C>A) is not predicted to affect splicing [Human Splicing Finder (20)]. However, this variant is rare [allele frequency 0.0007%, gnomAD v3.1 (21)] and has been previously reported (22). Thus, either one of these *ABCC8* changes may act as a recessive loss-of-function mutation and represent the first “hit” in this patient.

Based upon the experience at our institution, multiple focal lesions represent ~1% of all focal HI cases (*n* = 3/264) and 0.3% of all non-syndromic HI cases (*n* = 3/937) seen. The risk of focal HI in a fetus carrying a paternally inherited recessive K_ATP_ mutation (i.e., the risk of somatic paternal uniparental disomy within the pancreas) has been estimated to be 0.37% (1:270) (23). This may suggest that the risk of a second LOH event within the pancreas would also be 0.37%. The incidence of multifocal HI within focal HI cases seen at our institution (1.1%) is nearly three-fold higher than this estimate. The reason for this difference is not clear and may be explained by case ascertainment bias. Alternatively, this may indicate that the risk of pancreas-limited paternal uniparental disomy among carriers of recessive K_ATP_ channel mutations is higher than previously estimated.

These cases illuminate the pivotal role of collaboration across the fields of pediatric endocrinology, genetics, radiology, surgery, and pathology in optimizing care for patients with HI. Notably, while ^18^F-DOPA PET/CT accurately identified both pancreatic focal lesions in patient 2, this was not the case for patient 1 in whom the second focal lesion was identified intraoperatively owing to the surgical expertise and experience at our center. Through this multidisciplinary approach, both patients were cured.

## Conclusion

Rarely, patients with the focal form of HI may have more than one focal lesion. This occurs due to separate somatic events resulting in varying lengths of 11p15 LOH unique to each lesion. A multidisciplinary team approach, including specialists with extensive experience caring for patients with HI, plays a critical role in the effective diagnosis and treatment of congenital HI. The importance of this approach to patient care is especially highlighted by rare, atypical, and challenging cases as presented herein.

## Data Availability Statement

The relevant original contributions presented in the study are included in the article, further inquiries can be directed to the corresponding author.

## Ethics Statement

The studies involving human participants were reviewed and approved by Children's Hospital of Philadelphia Institutional Review Board. Written informed consent to participate in this study was provided by the participants' legal guardian/next of kin.

## Author Contributions

ER wrote the first draft of the manuscript. LM, KB, SB, HM, LB, AA, JK, TB, LS, NA, and KL contributed to the areas relevant to their expertise and edited the manuscript. DDDL conceptualized the work and edited the manuscript. All authors were involved in preparation of the manuscript.

## Conflict of Interest

The authors declare that the research was conducted in the absence of any commercial or financial relationships that could be construed as a potential conflict of interest.
